# Intermediate Outcomes of a Chronic Disease Self-Management Program for Spanish-Speaking Older Adults in South Florida, 2008–2010

**DOI:** 10.5888/pcd10.130016

**Published:** 2013-08-29

**Authors:** Michael A. Melchior, Laura R. Seff, Elena Bastida, Ahmed N. Albatineh, Timothy F. Page, Richard C. Palmer

**Affiliations:** Author Affiliations: Laura R. Seff, Elena Bastida, Richard C. Palmer, Ahmed N. Albatineh, Timothy F. Page, Florida International University, Miami, Florida.

## Abstract

**Introduction:**

The prevalence and negative health effects of chronic diseases are disproportionately high among Hispanics, the largest minority group in the United States. Self-management of chronic conditions by older adults is a public health priority. The objective of this study was to examine 6-week differences in self-efficacy, time spent performing physical activity, and perceived social and role activities limitations for participants in a chronic disease self-management program for Spanish-speaking older adults, Tomando Control de su Salud (TCDS).

**Methods:**

Through the Healthy Aging Regional Collaborative, 8 area agencies delivered 82 workshops in 62 locations throughout South Florida. Spanish-speaking participants who attended workshops from October 1, 2008, through December 31, 2010, were aged 55 years or older, had at least 1 chronic condition, and completed baseline and post-test surveys were included in analysis (N = 682). Workshops consisted of six, 2.5-hour sessions offered once per week for 6 weeks. A self-report survey was administered at baseline and again at the end of program instruction. To assess differences in outcomes, a repeated measures general linear model was used, controlling for agency and baseline general health.

**Results:**

All outcomes showed improvement at 6 weeks. Outcomes that improved significantly were self-efficacy to manage disease, perceived social and role activities limitations, time spent walking, and time spent performing other aerobic activities.

**Conclusion:**

Implementation of TCDS significantly improved 4 of 8 health promotion skills and behaviors of Spanish-speaking older adults in South Florida. A community-based implementation of TCDS has the potential to improve health outcomes for a diverse, Spanish-speaking, older adult population.

## Introduction

Hispanic people comprise the largest and fastest growing minority group in the United States (1) and disproportionately experience the incidence and negative health effects of chronic disease (2–4). The most common chronic diseases among the Hispanic population are diabetes, hypertension, liver disease, arthritis, respiratory diseases, stroke, cancer, and heart disease (3,4). Chronic diseases account for 6 of the top 10 leading causes of death for Hispanics (5), who disproportionately experience diabetes and cardiovascular diseases (4). Factors contributing to these disparities include language and cultural barriers, lack of access to preventive services, lack of insurance, and an increasing trend of chronic disease prevalence and comorbidity (6,7). Hispanics also face disparities in quality of, and access to, health care (8), which emphasizes the need to improve chronic disease self-management.

Because a large percentage of the US population is approaching age 65 and because health care costs continue to rise, self-management of chronic conditions by all older adults is a public health priority (9,10). Older adults who have 1 chronic disease are likely to develop more chronic diseases (11), and most older adults manage 2 or more chronic diseases simultaneously (12). Compared with other racial/ethnic groups, Hispanics report lower levels of chronic disease symptom management self-efficacy (13).

The Health Foundation of South Florida (HFSF) created the Healthy Aging Regional Collaborative (HARC) to offer evidence-based health promotion programs to the large older adult population in South Florida through community-based agencies. HARC provided a network for local community agencies to reduce start-up costs and other barriers to offering community-based health promotion programs, and to share best practices. 

Postulating that chronic disease self-management is a significant issue for the estimated 390,000 older Hispanic adults in South Florida (14), HARC chose to offer the Spanish-language chronic disease self-management program Tomando Control de su Salud (TCDS). The goal of TCDS is to improve disease management self-efficacy through skills mastery, modeling, reinterpretation of symptoms, and social persuasion techniques. TCDS, an evidence-based program (6), was developed to be culturally appropriate for Hispanic populations. Cultural beliefs play a significant role in health-related behaviors and attitudes (15). TCDS, which is based on self-efficacy theory proposed by Albert Bandura (16), addresses some of the significant issues identified in previous studies of Hispanic participants who did not show significant improvement in nutrition, food selection, food preparation, and menu planning after participating in the English-language Chronic Disease Self-Management Program (17). The association between self-efficacy and psychological well-being is supported by a study of Latinas who have a chronic disease (18). 

The purpose of this study was to examine whether TCDS improved symptom management self-efficacy, perceived social and role activities limitations, and time spent exercising, when implemented by community-based agencies through a large-scale collaborative effort in South Florida. The effort by HARC represented the first large-scale, community-based implementation of TCDS and was an example of Phase 4 translational research, the evaluation of health outcomes in a real-world setting. Since limited information is available on the translation of TCDS to practice settings, we focused on short-term program outcomes to evaluate effectiveness outside controlled trials. We hypothesized that program participants would show significant improvements over baseline scores for measures of self-efficacy, perceived social and role activities limitations, and time spent exercising when measured on the last day of workshop participation.

## Methods

### Setting and participants

HFSF requested proposals to participate in HARC. Through a peer-review process, 8 agencies were funded to deliver 82 TCDS workshops, using 82 instructors, at 62 sites throughout Broward and Miami-Dade Counties from October 1, 2008, through December 31, 2010. Two instructors led each class following the order and scripts in the leader's manual. Agencies offering TCDS were 6 community service agencies or health clinics that provide services for older adults, 1 hospital, and 1 county-level elder services department. Workshop sites were churches, nursing homes, community centers, residential community clubhouses, and health clinics. Agencies recruited participants from their client bases and the community through advertising and word of mouth. Program participants were self-selected and generally enrolled in the study before the start of the first workshop. The target population consisted of Spanish-speaking Hispanic adults aged 55 years or older who had at least 1 chronic disease.

Instructors were required to attend a 4-day (20-hour) program-specific training. New instructors were paired with an experienced instructor (19). Instructors were required to be health care professionals or peers and have experience managing a chronic disease. 

Maintaining fidelity is key to the successful translation of an evidence-based health promotion program (20). Random fidelity monitoring was conducted by using a log of proposed workshops and a random number generator to evaluate to what degree instructors were following the presentation order and scripts provided in the leader's manual. Fidelity monitoring was conducted on 12% (n = 10) of all TCDS workshops offered and included evaluation of sites, classroom environment, participant–instructor interaction, and delivery of program content using a standardized checklist.

### Intervention

Six 2.5-hour classes were offered once per week. TCDS uses didactic lectures, role play, brainstorming, written assignments, modeling, and goal setting to teach participants disease management skills, problem-solving techniques, critical thinking, and how to appropriately use available resources (21).

All participants completed an informed consent, a demographic survey, and a baseline survey before the start of the first session. At the end of the sixth and final session, participants in attendance completed a post-intervention survey. Instructors distributed the written surveys, which participants completed independently. Participants who were unable to read or write were assisted by instructors or agency staff. After the sixth session, agency staff entered TCDS participant data into an online database. The original forms were then sent to an independent evaluation team for data entry verification.

### Measures

We applied methods previously used to evaluate TCDS (6). Outcome measures included self-efficacy, perceived social and role activities limitations, and health behaviors. Examples of questions, response scales, and results of previous tests of reliability and validity are available elsewhere (22). No tests of reliability and validity were conducted by this study except for using Cronbach α values to evaluate the self-efficacy to manage disease scale and perceived social and role activities limitations scale.

Self-management behaviors were evaluated pre-intervention and post-intervention by using measures of exercise frequency and level of interference in social and daily activities by chronic disease symptoms. Other pre-intervention and post-intervention measures include a single item used to evaluate the amount of time per week spent performing stretching or strengthening exercises, and 2 items used to assess aerobic activity: time spent walking and time spent performing other aerobic activity. The measures assessing stretching or strengthening and aerobic exercises used a Likert response scale (0 = none, 5= more than 3 hours per week). The 4 items used to measure perceived social and role activities limitations used a Likert response scale (0 = almost totally, 4 = not at all). Participants were asked to rate how much their health interfered with the following: normal social activities with family and friends, hobbies or recreational activities, household chores, errands and shopping. Cronbach's α for this scale was 0.93. An average across all answered items was calculated.

Measures of confidence across multiple aspects of disease management — managing disease, managing emotions, communicating with a physician, and using mental and physical techniques learned from the program to manage symptoms — were assessed at baseline and post-intervention and used to evaluate self-efficacy using a Cantril ladder response scale (1 = not at all confident, 10 = totally confident). Three items were used to measure self-efficacy to manage disease. These items asked participants to rate self-efficacy to keep health problems, discomfort, and fatigue from interfering with daily activities. To be included in analysis, participants were required to answer all 3 items. Cronbach's α for the scale was 0.94. An average across 3 items was calculated. Single items were used to measure self-efficacy to manage emotions, self-efficacy to communicate with a physician, and self-efficacy to use techniques learned in class.

Measures that describe participant characteristics and attitudes at baseline include information on sex, age, race/ethnicity, income level, highest education level, marital status, disability status, household number, and county of residence in South Florida. A single item taken from the National Health Interview Survey (23) assessed self-rated health with response options of poor, fair, good, very good, and excellent. Level of pain, fatigue, shortness of breath, and frustration in the previous 2 weeks were measured using a modified visual-numeric scale. Using 10 histograms of gradually different heights and shading intensities, the response scale ranged from 0 being none to 10 being extreme. The number of days out of the past 30 that participants’ physical and mental health was “not good” and the number of days out of the past 30 that participants’ health hindered involvement in their usual activities were also reported (22). A 3-item scale was used to assess physician–patient communication, which included items regarding frequency of preparing a question list, asking questions, and discussing personal problems with a physician using a Likert response scale (0 = never, 5 = always). Cronbach’s α for this scale was 0.74. To evaluate health care use, participants were asked to report the number of visits to physicians, visits to emergency departments, hospitalizations, and nights spent in a hospital during the past 6 months. Each of these measures has been used in previously published studies (22,24). None of these measures were repeated at post-test.

### Data analysis

Participant data were extracted from an online database; 1,186 participants enrolled in the program. Participants younger than 55 years or missing data on age (n = 160) or those not having completed both a baseline and post-intervention survey (n = 344), were removed from the data set, leaving 682 to be included in analysis. Analysis was performed using SPSS version 17 (SPSS Institute, Inc, Cary, North Carolina). Data were cleaned of outliers and values outside possible response limits. Counts, means, and standard deviations (SDs) were obtained using frequency and descriptive data reports. One-way analysis of variance (ANOVA) was used to determine if outcome differences existed based on demographic characteristics and baseline health status measures. The Bonferonni method was used to determine whether significant differences existed for multiple comparisons. The general linear model was chosen to assess within-subject changes in outcome measures (self-efficacy, health behaviors, and social and role activities) at baseline and 6 weeks, because it is able to control for multiple covariates simultaneously (25). On the basis of findings from ANOVA, we controlled for both delivering agency and general health (26) at baseline. Controlling for agency effect was chosen, because stratification to lower levels would have required a larger sample (27). Power analysis was not conducted before the start of the study.

## Results

From October 1, 2008, through December 31, 2010, 682 participants attended at least 1 session of TCDS and met the inclusion criteria to be included in analysis. No significant differences were observed in demographic and baseline values between participants included and those excluded from analysis.

Average participant age was 76 (Table 1). Most participants were female (83%), were living in Miami-Dade County (78%), were single or not partnered (60%), had an annual household income of less than $15,000 (63%), and had less than a high school education (38%). Participants attended an average of 4.9 (SD, 1.4) sessions out of 6 and reported an average of 2 chronic diseases; 25.2% reported 3 or more. All participants identified as Hispanic; no additional data on Hispanic subculture (eg, country of ancestry) were collected.

**Table 1:** Demographic and Health Status Characteristics of Participants, Tomando Control de su Salud, South Florida, 2008–2010^a^

Characteristic	All Eligible Participants (N = 682)
**Mean age, y (SD)**	76.4 (8.7)
**Mean no. of chronic diseases (SD)**	2.0 (1.1)
**Sex, no. (%)**	
Female	566 (83.0)
Male	107 (15.7)
**County, no. (%)**	
Broward	138 (20.2)
Miami-Dade	533 (78.2)
**Marital status, no. (%)**	
Married/partnered	261 (38.3)
Single/not partnered	407 (59.7)
**Disabled, no. (%)**	
Yes	63 (9.2)
No	274 (40.2)
**Annual household income, no. (%)**	
<$15,000	428 (62.8)
≥$15,000	51 (7.5)
**Number in household (%)**	
Lives alone	415 (60.9)
Lives with others	267 (39.1)
**Education level, no. (%)**	
Less than high school	262 (38.4)
High school graduate/GED	193 (28.3)
Some college	79 (11.6)
College graduate	93 (13.6)
**Health status, mean (SD)**	
Self-rated health^b^	3.2 (0.99)
Poor physical health days^c^	5.8 (9.3)
Poor mental health days^c^	4.7 (9.0)
Days where activities were prevented^c^	3.5 (7.9)
Communication with physician^d^	2.5 (1.5)
MD visits^e^	2.8 (2.5)
ER visits^e^	0.2 (0.8)
Times hospitalized^e^	0.2 (1.0)
Days in hospital^e^	0.5 (3.0)
Level of fatigue^f^	3.1 (3.0)
Level shortness of breath^f^	1.9 (2.7)
Level of pain^f^	3.5 (3.3)
Level of frustration^f^	2.0 (2.7)

Abbreviations: SD, standard deviation; GED, general educational diploma.

a Cells may not add up to the study sample total because surveys were self-report and participants were encouraged, but not required, to answer all questions.

b Response scale of 1 = excellent to 5 = poor.

c Number of days out of the past 30.

d A 3-item scale, which included items asking frequency of preparing a question list, asking questions, and discussing personal problems with a physician. Response scale of 0 = never to 5 = always.

e Number of times in the past 6 months.

f Response scale of 0 = none to 10 = extreme, using 10 corresponding histograms of different heights and shading intensities.

There were significant improvements at 6 weeks (post-test) in 4 of the 8 health behavior measures (Table 2). Participants' self-efficacy to manage symptoms significantly increased 19.3% (change = 1.30; SD = 2.94; *P* = .006) between baseline and 6 weeks. Time spent walking increased 38.5% (change = 0.55; SD = 1.40; *P* = .02), and time spent performing other aerobic activity increased 104.7% (change = 0.45; SD = 1.39; *P* = .005). Participants’ perceived social and role activities limitations improved 1.6% (change = .05; SD = 1.28; *P* = .001). ANOVA with Bonferonni corrections found no significant differences in outcomes within demographic variables (eg, male vs female).

**Table 2:** Change in Participant (N = 682) Outcomes From Baseline to 6 Weeks, Tomando Control de su Salud, South Florida, 2008–2010^a^

Outcome	n	Score at Baseline	Score at 6 Weeks	Change in Score	*P* Value^b^

		Mean (SD)			
Self-efficacy to manage disease^c^	664	6.75 (2.66)	8.05 (2.16)	1.30 (2.94)	.006
Self-efficacy to manage emotions^c^	637	6.66 (2.88)	8.11 (2.40)	1.45 (3.30)	.16
Self-efficacy to use mental and physical techniques to manage symptoms^c^	641	6.02 (3.27)	8.21 (2.25)	2.19 (3.64)	.79
Self-efficacy to communicate with physician^c^	643	7.90 (2.60)	8.73 (2.12)	0.83 (2.81)	.48
Social/role activities limitations^d^	655	3.15 (1.05)	3.20 (1.08)	0.05 (1.28)	.001
Time spent stretching^e^	639	1.08 (1.20)	1.77 (1.29)	0.69 (1.54)	.06
Time spent walking^e^	599	1.43 (1.35)	1.98 (1.36)	0.55 (1.40)	.02
Time spent performing other aerobic activity^e^	575	0.43 (0.95)	0.88 (1.33)	0.45 (1.39)	.005

Abbreviation: SD, standard deviation.

a Cells may not add up to the study sample total because surveys were self-report and participants were encouraged, but not required, to answer all questions.

b *P* value statistic from the general linear model.

c Response scale of 1= not at all confident to 10 = totally confident.

d Response scale of 0 = almost totally to 4 = not at all.

e Response scale of 0 = none to 5 = more than 3 h/week.

Sessions observed for fidelity monitoring lasted an average of 1 hour and 46 minutes and had an average of 9 attendees. Results of fidelity monitoring found a high adherence rate for program content and delivery in the 10 (12%) workshops observed. No deviations from scripted content and delivery methods were observed. The most often identified fidelity issue (50%) was the presence of distractions during class; many workshops were conducted in common areas, and disruptions were the result of site clients or personnel passing through the classroom or making noise.

## Discussion

The objective of this study was to test the hypothesis that significant improvements at 6 weeks would be observed for measures of self-efficacy, health behavior, and perceived social and role activities limitations. Four of the measured outcomes showed significant differences from baseline to 6 weeks post-intervention. These measures were self-efficacy to manage symptoms, time spent walking, time spent performing other aerobic activity, and perceived social and role activities limitations.

Our results for participant self-efficacy to manage symptoms support 2 studies evaluating differences between baseline and 4 and 12 months (6,28). Physical activity is important in managing chronic disease, because it has been linked to a reduction in symptom severity and an improved perception of overall health (22,29). The findings of our study for time spent performing other aerobic activity support 2 studies evaluating differences in minutes spent performing aerobic activity between baseline and 4 and 12 months (6,28). These studies converted the Likert scale completed by participants by assigning minute values that fall half-way between the ranges provided (eg, 1 to 3 hours equals 120 minutes). Maintenance of social activity and roles (activities of daily living) are important for older adults because they can reduce depression, reduce the risk of disability, and slow cognitive decline (30). Our finding of improvement in perceived social and role activities limitations supports findings reported by Lorig and colleagues, who evaluated differences between baseline and at 4 and 12 months (6,28). Compared with past findings, our magnitude of change seems small. However, because our study evaluated outcomes only at 6 weeks, compared with others that evaluated outcomes at 4 and 12 months, the impact of the program may not have had time to reach full potential benefit. 

We evaluated the effectiveness of TCDS when implemented at the community level, by community agencies. Previous self-management studies have shown that similar health behavior changes, when sustained, continue to affect health positively and reduce use of health care services (31). Future evaluations at longer-term intervals (ie, at 6 or 12 months) may identify at what point program benefits are no longer retained and where a booster course may be warranted, issues that were not explored in the original research trials. HARC was a community-based implementation and not a controlled research study, so measures were not collected at these longer-term intervals when access to participants was no longer readily available. Future research may also further evaluate the effectiveness of TCDS when delivered to subgroups of the Hispanic community. Although TCDS was specifically developed to be culturally appropriate for Spanish speakers, the diverse subgroups in the Hispanic community may benefit from additional cultural tailoring, as would participants at different stages in the acculturation process (7).

Our study had limitations. Because some participants were recruited from nursing homes, adult day care centers, and sites having a standing history of clients, such as activity centers or health care clinics, the sample may not be representative of the general population. Because participants were self-selected, bias may have been introduced to both the sample and the results, based on the participants’ ability and eagerness to learn. The self-administration of surveys could introduce report and recall biases, because responses were not verified. Inherent with self-reporting and implementation in a community setting, many fields had missing data; it was not possible to contact participants to complete the missing fields. Results may have been influenced by other factors during the 6 weeks over which the workshop was offered, such as visits to health care providers. The study design did not allow for identification of the effectiveness of individual program components.

Our study also had several strengths. By using an evidence-based program with validated measures, we are confident that the link between program participation and outcomes is a causal one. Use of existing validated measures allowed us to ensure that we were measuring the concepts we set out to measure. Despite potential sample bias previously mentioned, the diversity among the agencies delivering TCDS and participants increased the generalizability of results, because study implementation resembles what can be expected of future community-based translations. Agencies offering TCDS benefited from being members of HARC, because it covered program licensing costs, coordinated instructor trainings, advertised workshop offerings, and led monthly conference calls to discuss implementation concerns faced by the agencies. Sustainability plans indicate that agencies will adopt TCDS fully and seek continued funding on their own.

Because Hispanics are disproportionately affected by incidence of chronic diseases, efforts should be made to decrease the disparities in prevalence and severity. Findings from this study show that evidence-based health promotion programs targeting older Hispanics with 1 or more chronic conditions increase participants’ ability to control important elements of disease management. Participants improved across all measures, and some improvements were significant. Because some measures did not improve significantly, program adaptation specific to the culture and needs of the Hispanic subgroups of South Florida may be warranted. Research studies involving Hispanic populations found significantly different outcomes based on subgroup (eg, Cuban, Mexican, Chilean) (7,15). Although these groups are all considered Hispanic, the differences in culture, beliefs, and attitudes warrant culturally competent programs. Formative research with these populations can help tailor the TCDS program to be more culturally competent. Additional research should also evaluate the effectiveness of TCDS when translated by community agencies and in different Hispanic subgroups throughout the United States.
